# PReS13-SPK-1182: Six years of eu paediatric regulation - what was achieved for paediatric rheumatology

**DOI:** 10.1186/1546-0096-11-S2-I15

**Published:** 2013-12-05

**Authors:** R Vesely

**Affiliations:** 1 Paediatric Medicines, European Medicines Agency, London, UK

## 

EU Paediatric Regulation was adopted by the European Parliament and the Council in December 2006. First meeting of the Paediatric Committee took place in July 2007.

1. Paediatric investigation Plans (PIPs)

In 2007-2013 The European Medicines Agency (EMA) and its Paediatric Committee have agreed more than 600 Paediatric Investigation Plans (PIPs) with pharmaceutical companies, to provide data on the efficacy and safety of medicines for diseases of children.

· For treatment of JIA PIP was agreed for the following substances: abatacept, adalimumab, anakinra, anti-BAFF antibody, anti-IL-6 antibody, anti-IL17 antibody, anti-IL-17A antibody, apremilast, baricitinib, canakinumab, certolizumab, denosumab, etanercept, givinostat, golimumab, olokizumab, sarilumab, tocilizumab, tofacitinib and ustekinumab.

· For treatment of SLE in children PIP was agreed for anti-BAFF, belimumab and epratuzumab.

· For treatment of CAPS PIP was agreed for anakinra, anti-IL-1beta antibody and canakinumab.

2. Clinical trials

More paediatric clinical trials were done (data from EudraCT, accessible at the European Clinical Trials Register):

3. New authorised indications for children

· 12/11/2009: canakinumab for treatment of CAPS.

· 25/08/2008: adalimumab - treatment of active polyarticular juvenile idiopathic arthritis in adolescents from 13 to 17 years of age.

· 20/01/2010 abatacept - treatment of moderate to severe active polyarticular juvenile idiopathic arthritis in paediatric patients 6 years of age and older.

· 18/03/2011 adalimumab - treatment of active polyarticular juvenile idiopathic arthritis in the paediatric population aged from 4 to 12 years.

· 01/08/2011: tocilizumab - treatment of active systemic juvenile idiopathic arthritis (sJIA) in patients 2 years of age and older.

· 24/08/2011: etanercept - polyarticular juvenile idiopathic arthritis (JIA) from the age of 2 years.

· 24/08/2012: etanercept - treatment of RF+ and RF- polyarthritis, extended oligoarthritis from 2 years of age, psoriatic arthritis and ERA from 12 years of age.

· 17/01/2013 - adalimumab - treatment of active polyarticular juvenile idiopathic arthritis in the paediatric population aged from 2 to 4 years old.

· 25/04/2013 - tocilizumab - treatment of polyarthritis (RF+, RF- and extended oligoarthritis) from 2 years old (EC decision pending at the time of abstract submission).

4. A network of paediatric research networks

Enpr-EMA works by fostering collaboration from within and outside the European Union (EU), including between members, patients associations, academia and the pharmaceutical industry.) PRINTO and JSWG of PRES are members of Enpr-EMA.

5. Expert meetings at EMA:

Paediatric rheumatology (2009, 2010), Gastroenterology and rheumatology (2010).

6. Development of scientific guidelines (JIA, SLE, GIOP).

7. Pharmacovigilance

ENCEPP - (PharmaChild protocol agreed).

## Disclosure of interest

None declared.

